# Diagnostic value of cerebrospinal fluid human epididymis protein 4 for leptomeningeal metastasis in lung adenocarcinoma

**DOI:** 10.3389/fimmu.2024.1339914

**Published:** 2024-01-18

**Authors:** Xiangyu Li, Kun Chen, Jie Li, Xuemei Tang, Haoyu Ruan, Ming Guan

**Affiliations:** ^1^ Department of Laboratory Medicine, Huashan Hospital, Fudan University, Shanghai, China; ^2^ Department of Laboratory Medicine, The First Affiliated Hospital of Nanjing Medical University, Nanjing, China

**Keywords:** cerebrospinal fluid, human epididymis protein 4, CEACAM6, lung adenocarcinoma, leptomeningeal metastasis

## Abstract

**Background:**

The diagnosis of lung adenocarcinoma (LUAD) leptomeningeal metastasis (LM) remains a clinical challenge. Human epididymis protein 4 (HE4) functions as a novel tumor biomarker for cancers. This study aimed to assess the diagnostic value of cerebrospinal fluid (CSF) HE4, and combined with CEACAM6, for LUAD LM.

**Methods:**

The CSF HE4 protein level was measured in two independent cohorts by electrochemiluminescence. Test cohort included 58 LUAD LM patients, 22 LUAD patients without LM (Wiot-LM), and 68 normal controls. Validation cohort enrolled 50 LUAD LM patients and 40 normal controls, in parallel with Wiot-LM patients without brain metastases (19 Wiot-LM/BrM patients) or with BrM (26 BrM patients). The CSF level of CEA, CA125, CA153, CA199, CA724, NSE and ProGRP of these samples was measured by electrochemiluminescence, whereas the CSF CEACAM6 level was detected by enzyme-linked immunosorbent assay (ELISA). In addition, the serum level of these biomarkers was detected by same method as CSF.

**Results:**

The level of HE4 or CEACAM6 in CSF samples from LUAD LM patients was significantly higher than those from normal controls and Wiot-LM patients. The HE4 or CEACAM6 level in CSF was higher than that in serum of LM patient. The CSF HE4 or CEACAM6 level for distinguished LM from Wiot-LM showed good performance by receiver-operating characteristic analysis. The better discriminative power for LM was achieved when HE4 was combined with CEACAM6. In addition, the CSF HE4 and CEACAM6 level showed little or no difference between Wiot-LM/BrM and BrM patients, the BrM would not significantly influence the HE4 or CEACAM6 level in CSF. The diagnostic power of CSF CA125, CA153, CA199, CA724, NSE and ProGRP for LUAD LM were not ideal.

**Conclusion:**

The combination with HE4 and CEACAM6 has the promising application for the diagnosis of LUAD LM.

## Introduction

Almost 3–5% of advanced non-small-cell lung cancer (NSCLC) patients suffer from leptomeningeal metastasis (LM), and the incidence has increased as a result of new molecular therapies improving outcomes of patients (1). The poor prognosis of lung adenocarcinoma (LUAD) LM is in part due to the delayed diagnosis. Diagnosis of LM depends on clinical symptoms, radiographic imaging, abnormal neurological and cerebrospinal fluid (CSF) findings. The positive cytologic assessment is gold standard for LM diagnosis, but sensitivity is as low as 45%-67% in initial CSF analysis and increase to 80% to 89% by repeated lumbar puncture (2). The CSF of LM patients show increased intracranial pressure, cell numbers, and protein level, which could help diagnosis LM. Tumor biomarkers with favorable diagnostic value may provide valuable clues for LM, however the CSF tumor markers for LUAD LM diagnosis have been unclear. Our previous study has discovered high CEACAM6 level in CSF and confirmed CSF CEACAM6 as a crucial biomarker for LM diagnosis (3). In addition, we discovered the CSF-CTCs of LUAD LM patients showed higher expression of gene WFDC2 which encoded secrete protein HE4 (4). Therefore, we tried to detect the CSF HE4 concentration and identify it whether could serve as a biomarker for LUAD-LM patients.

HE4 is secreted extracellularly into the bloodstream and frequently upregulated in many malignancies as a tumor biomarker. HE4 was commonly used in clinic as its unique roles in ovarian cancer, not only for diagnosis, but also for prognosis and recurrence (5). In lung cancer, serum HE4 can be used as the diagnosis biomarker for lung cancer (LC), particularly for early-stage LC (6), and correlates with poor prognosis, especially in Asian patients (7). HE4 in the pleural fluid can help differentiate malignant effusions from benign effusions (8, 9). So far, research associated with HE4 in lung cancer has been mainly focused on serology and pleural effusion, few studies have investigated the clinical significance of HE4 in CSF.

In this study, we aimed to compare CSF HE4 levels between LUAD LM patients and LUAD patients without LM or normal controls, determine the diagnostic value of CSF HE4 or combined with CEACAM6 for LUAD LM.

## Materials and methods

### Study participants and sample collection

CSF and paired serum samples were recruited from Huashan hospital, Fudan university. Test cohort included 58 LUAD LM patients, 22 LUAD patients without LM (Wiot-LM), and 68 normal controls. In validation cohort, the Wiot-LM group were further divided into Wiot-LM patients without brain metastases (Wiot-LM/BrM), and Wiot-LM patients with BrM (BrM). In total, validation cohort enrolled 50 LUAD LM patients, 19 Wiot-LM/BrM patients, 25 BrM patients, and 40 normal controls. LUAD LM patients were diagnosed based on positive CSF cytology. Wiot-LM group was LUAD patients, who showed non-detection of tumor cells in the CSF by cytomorphological examination and typical radiographic features at the time of CSF sample collection and the next 6 months of follow-up. CSF samples enrolled in control group were collected from patients who is suspected of having neurological disorders (neuroinfectious diseases and neurodegenerative and neuroinflammatory diseases) but confirmed normal by CSF examination including general, chemical composition, cytology, pathogenic microorganisms examination.

This study was approved by the Institute Research Ethics Committee of the Cancer Center of Huashan hospital. Informed consent was obtained from participants.

### Laboratory methods

The CSF (3000 g/min, 5min) and serum (1000 g/min, 5min) samples were centrifuged to remove cell pellet, and the supernatant were collected and stored at −80°C prior for analysis.

The HE4 (cat:130201525M), CA125 (cat: 130201009M), CA153 (cat:130201010M), CA199 (cat: 130201011M), CA724 (cat: 130201015M), NSE (cat:130201016M), ProGRP (cat: 130201523M) and CEA (cat: 130201003M) concentrations were not diluted and investigated using a MAGLUMIG X8 chemiluminescent micro-particle immunoassay (CMIA) Analyzer (Briefed as Snibe Co.,Ltd.) according to the instructions. If samples concentrations were over the limit, the level was recorded as the upper limit value in the study. The test was performed by sandwich chemiluminescence immunoassay. The sample was incubated by anti-HE4 monoclonal antibody magnetic microbeads and then the precipitation was washed one time. Subsequently, another anti-HE4 monoclonal antibody labeled by ABEI (N-(4-Aminobutyl)-N-ethylisoluminol) was added to form sandwich complexes. After precipitation in a magnetic field, the supernatant was decanted and then a wash cycle is performed. Lastly, a chemiluminescent reaction was initiated and the light signal was measured.

The CEACAM6 level were measured by enzyme-linked immunosorbent assay ELISA (Sino Biological, Cat: SEKA10823) and the dilutions were 0.1% BSA. Although the kit is for research use only and is not for use in diagnostic or therapeutic procedures, the performance characteristics (Precision, Accuracy, Specificity, and Linearity have been analyzed).

### Single cell data analysis

Single-cell RNA-seq data (Smart-seq2 method) of CSF-CTCs from five LUAD LM patients (P1, P2, P4, P6 and P7) and CSF immune cells from three normal samples (N1, N2, N3) (4), single-cell RNA-seq data (10× genomics method) of CSF cells from two NSCLC LM patients (10) were enrolled in the study to analyzed the WFDC2 mRNA expression in different CSF cells. In addition, single-cell RNA-seq data (10× genomics method) of cells of brain metastasis (BrM) from 10 LUAD patients (NS02, NS03, NS04, NS06, NS07, NS12, NS13, NS16, NS17, NS19) (11) were enrolled to compare the WFDC2 mRNA expression in tumor cells and other cells in BrM. The gene expression matrix and cell types identification in these single-cell datasets have been access on published articles, and the R package Seurat were used to demonstrate the normalized HE4 expression counts of known cell types.

### Immunocytochemical staining for HE4

The CSF cells were centrifuged on slides for HE4 protein immunocytochemical staining. The steps of immunocytochemical staining were as follows: 1) Block the endogenous peroxide enzymes using 0.3% hydrogen peroxide (25°C,15 min), then wash with distilled water for 3 min. 2) Do the antigen retrieval in the EDTA buffer (EnVision FLEXTARGET RETRIEVAL SOLUTIONHIGH) of pH 9.0 for 17 min at 97°C (Dako PTlink), make the slides cool down to room temperature naturally and wash three times with DaKO EnVision™ FLEX wash buffer. 3) Take the normal sheep serum to block the nonspecific antigens at 25°C for 15 min. 4) Incubate with primary antibody against HE4 (MAB-0826, Maixin Biotech, Fujian, China) for 1h at 37°C and wash three times with a PBS buffer for 3 min. 5) Incubate with anti-rabbit/mouse HRP secondary antibodies (MaxVision DAB, Maixin Biotech, Fujian, China) for 1h at 37°C and wash three times with PBS. 6) Add the DAB (diamino-benzidine) solution (MaxVision DAB, Maixin Biotech, Fujian, China) to observe the coloration for 15 min and stop the reaction with running water. 7) Perform nuclear stain by haematoxylin for about 2 min and remove excess staining solution with tap water. 8) dehydrate by gradient alcohol (75%-80%-95%-100%-100%) for seconds and seal the slides with a neutral gum.

### Statistical analyses

Statistical analyses were performed with SPSS 22.0 (IBM Corp) and GraphPad Prism Software (GraphPad Inc). p<0.05 was considered statistically significant. For measurement data skewed, nonparametric tests the Mann-Whitney test or Wilcoxon rank-sum test was used to compare the unpaired data or paired data between the two groups. Receiver-operating characteristic (ROC) curves were plotted to assess the diagnostic efficiency by the GraphPad Prism Software, the area under the curve (AUC) including 95% confidence interval (CI) values were determined.

## Results

### Clinicopathological features of the patients

The characteristics of patients in control, Wiot-LM and LM groups are summarized in **Table 1**.

**Table 1:** Clinical characteristics and diagnosis of patients enrolled in the test cohort and validation cohort.

	Patients (n)	Male/female (n)	Age, Mean ± SEM (yr)	Age range (yr)
Test cohort				
***Control***	*68*	*35/33*	*46.66 ± 1.74*	*18-84*
***Wiot-LM***	*22*	*14/8*	*64.05 ± 1.66*	*50-81*
***LM***	*58*	*19/39*	*56.00 ± 1.29*	*32-76*
Validation cohort				
***Control***	*40*	*18/22*	*62.92 ± 1.88*	*45-82*
***Wiot-LM/BrM***	*19*	*11/8*	*60.06 ± 2.45*	*44-77*
***BrM***	*26*	*16/10*	*65.48 ± 2.26*	*45-81*
***LM***	*50*	*28/22*	*63.84 ± 1.89*	*38-79*

Test Cohort. The patients in control group comprised 35 males and 33 females with an average age of 46.66 ± 1.74 years. 58 LUAD patients in LM group consisted of 19 males and 39 females aged 32 to 76 years with an average age of 56.00 ± 1.29 years. Of 22 patients in With-LM group, 14 were males and the average age was 64.05 ± 1.66 ranging from 50 to 81 years.

Validation Cohort. The patients in control group comprised 18 males and 22 females with an average age of 62.79 ± 1.83 years. 50 LUAD patients in LM group consisted of 28 males and 22 females aged 38 to 79 years with an average age of 63.84 ± 1.89 years. Of 19 patients in With-LM/BrM group, 11 were males and the average age was 60.06 ± 2.45 ranging from 44 to 77 years. Of 26 patients in BrM group, 10 were females and the average age was 65.48 ± 2.26 ranging from 45 to 81 years.

### CSF-CTC of LM and BrM tumor cells expressing high WFDC2 mRNA level in lung cancer at single cell level

**Figure 1:**
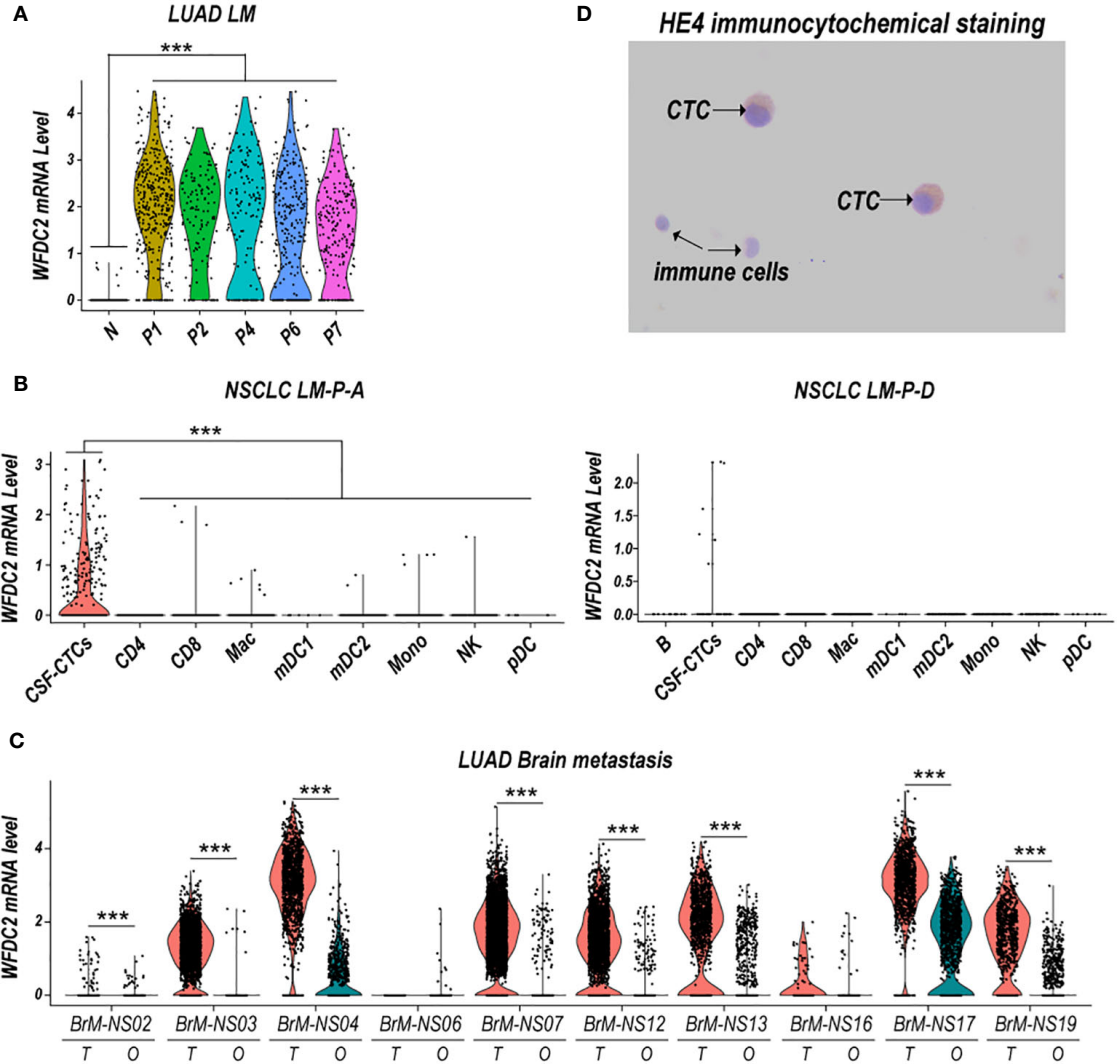
WFDC2 (encoding HE4) mRNA expression in tumor cells of lung cancer LM and BrM. **(A)** Violin plots of WFDC2 mRNA expression in normal CSF cells (N) from three normal CSF samples (N1, N2 and N3) and cerebrospinal fluid circulating tumor cells (CSF-CTCs) of five lung adenocarcinoma (LUAD) leptomeningeal metastasis (LM) patients (P1, P2, P4, P6 and P7). **(B)** Violin plots of WFDC2 mRNA expression in CSF-CTCs and various immune cells of non-small cell lung cancer LM patient A (NSCLC LM-P-A, left) or patient D (NSCLC LM-P-D, right) CSF samples. Cluster key: pDC, plasmacytoid dendritic cells; mDC1, myeloid DC type 1; mDC2, myeloid DC type 2; Mono, monocytes; Mac, macrophages; CD8, CD8+ T cells; CD4, CD4+ T cells; NK, natural killer cells; B, B cells. **(C)** Violin plots of WFDC2 mRNA expression in tumor cells (T) and others cells (O) in brain metastases (BrM) of 10 LUAD patients (NS02, NS03, NS04, NS06, NS07, NS12, NS13, NS16, NS17, NS19). ***p-value <0.001, Wilcoxon rank-sum test. **(D)** The immunocytochemistry of HE4 in CSF cells of a LUAD LM patient. Magnification 400×.

Single cell RNA-seq data showed that circulating tumor cell (CSF-CTCs) from seven NSCLC-LM patients (P1, P2, P4, P6, P7, P-A and P-D) had higher level of WFDC2 mRNA (encoding HE4) than CSF immune cells (**Figures 1A, B**). We analyzed tumor cells from 10 patients with LUAD brain metastasis (BrM), and discovered WFDC2 mRNA level was also higher in tumor cells compared to other cells of BrM tumor (**Figure 1C**). The number of tumor cells and other type cells of each sample in **Figure 1** has been shown in **Supplementary Table 1**. In addition, as the **Figure 1D** showed, CSF-CTCs had larger cell body and nuclear, and more abundant cytoplasm than CSF immune cells. The immunohistochemistry staining showed most CSF-CTCs had higher expression of HE4 protein in cytoplasm (**Figure 1D**). Therefore, the CSF-CTCs could secret high HE4 level into CSF of LUAD LM patients and HE4 from immune cells would not influence the CSF HE4 level.

### HE4 level was elevated in LUAD LM patients in test cohort

**Figure 2:**
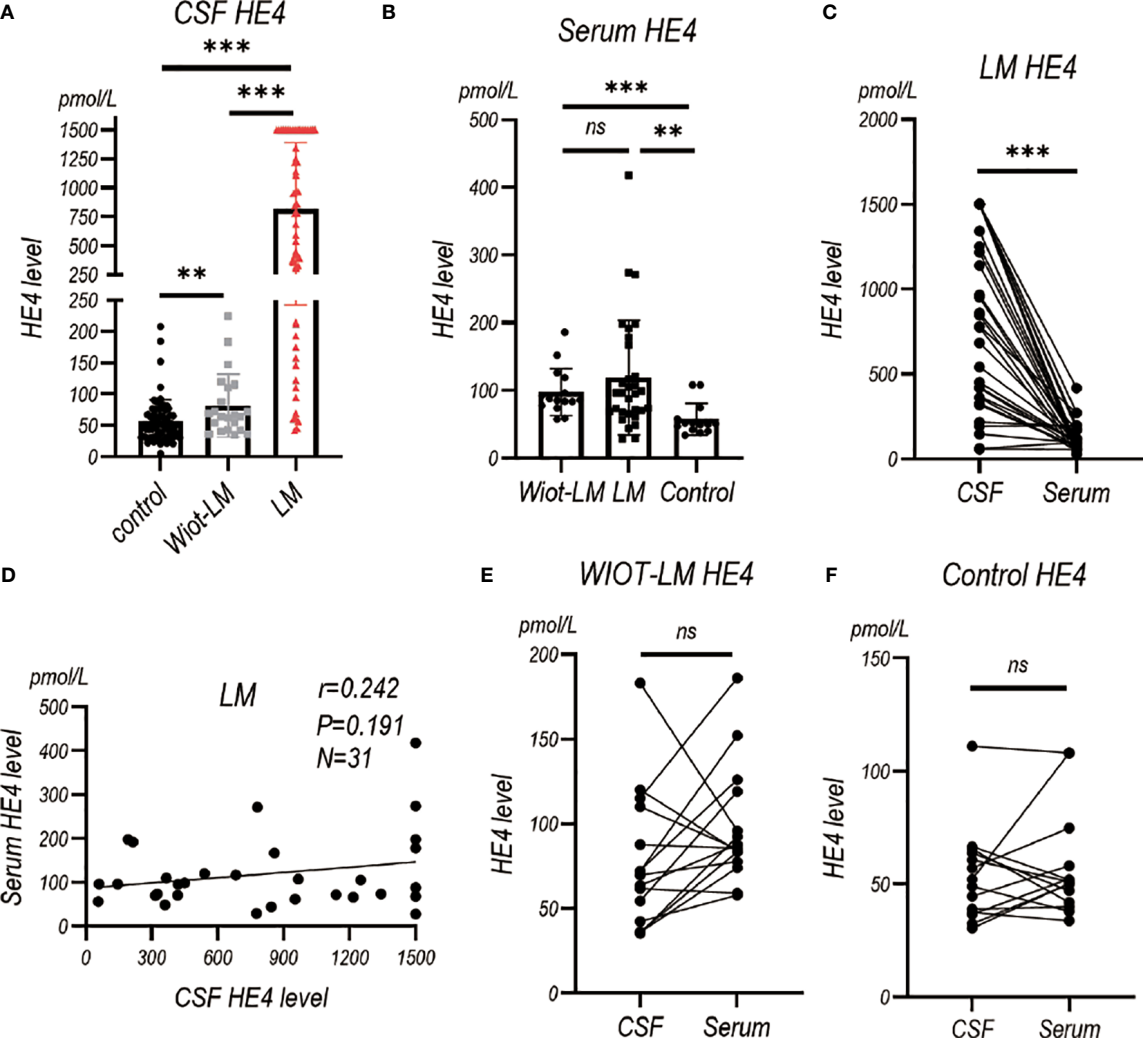
The HE4 level in CSF and Serum in test cohort. **(A)** The CSF HE4 level in normal control samples (Control, N=68), and LUAD patients with leptomeningeal metastasis (LM, N=58) or without LM (Wiot-LM, N=22). **(B)** The serum HE4 level in Control (N=14), Wiot-LM (N=15) and LM (N=31) patients. **(C)** The paired CSF and serum HE4 level in LM patients (N=31). **(D)** The correlation between CSF and serum HE4 level (N=31) in LM patients. **(E, F)** The paired CSF and serum HE4 level in Wiot-LM (E, N=15) and Control (F, N=14) patients. ***p-value <0.001, **p-value <0.01, ns, not statistically significant.

To assess the diagnostic value of HE4 in the CSF of LUAD LM patients, electrochemiluminescence method was performed to detect HE4 level in CSF (**Table 2**). An average of 816.50 ± 75.36 pmol/L of CSF HE4 protein were detected in LUAD LM patients, more than 10-fold higher than that in the Wiot-LM (81.80 ± 10.64 pmol/L) and control (55.91 ± 4.26 pmol/L) groups (**Figure 2A**). However, there was no difference in serum HE4 level between Wiot-LM and LM group (**Figure 2B**). For LM patients, the HE4 level in CSF was significantly higher than that in serum (**Figure 2C**) and had no correlation with serum HE4 (**Figure 2D**), whereas for patients in Wiot-LM and control group, HE4 level showed no difference between CSF and serum (**Figures 2E, F**). LUAD LM patients showed higher CSF HE4 than Wiot-LM and control patients, and HE4 serum level would not significantly influence CSF level.

**Table 2:** The CSF or serum concentrations of tumor markers in patients of Control, Wiot-LM, and LM groups in test cohort and validation cohort.

Test cohort																
Tumor markers	CSF								Serum							
	Control		Wiot-LM		LM				Control		Wiot-LM		LM			
	Number	Mean ± SEM (Min-Max)	Number	Mean ± SEM (Min-Max)	Number	Mean ± SEM (Min-Max)			Number	Mean ± SEM (Min-Max)	Number	Mean ± SEM (Min-Max)	Number	Mean ± SEM (Min-Max)		
***HE4 (pmol/L)***	*68*	*55.91 ± 4.26* *(5.00-208.00)*	*22*	*81.80 ± 10.64* *35.40-224.00)*	*58*	*816.50 ± 75.36* *41.90-1500.00)*			*14*	*57.5 ± 6.30* *33.80-108.00)*	*15*	*97.69 ± 8.96* *57.90-186.00)*	*31*	*119.00 ± 15.18* *(28.10-418.00)*		
***CEACAM6*** ***(ng/ml)***	*49*	*1.60 ± 1.40* *(0.02-68.66)*	*22*	*1.86 ± 0.71* *(0.02-11.82)*	*45*	*76.64 ± 6.15* *2.27-129.20)*			*/*	*/*	*/*	*/*	*/*	*/*		
***CEA (U/ml)***	*66*	*1.17 ± 0.540* *(0.20-34.64)*	*22*	*13.53 ± 7.20* *0.20-124.70)*	*58*	*274.30 ± 110.80* *(0.20-4866.00)*			*17*	*2.29 ± 0.44* *(0.32-6.07)*	*19*	*14.35 ± 5.11* *1.34-80.09)*	*35*	*186.50 ± 80.65* *(2.33-2671.00)*		
***CA125 (U/ml)***	*/*	*/*	*22*	*1.73 ± 0.79* *0.50-16.20)*	*29*	*234.00 ± 116.40* *(0.60-3137.00)*			*/*	*/*	*19*	*30.67 ± 9.34* *(9.62-147.70)*	*31*	*131.80 ± 55.18* *9.51-1165.00)*		
***CA153 (U/ml)***	*/*	*/*	*22*	*1.24 ± 0.07* *(0.60-1.60)*	*47*	*10.99 ± 3.60* *1.00-125.40)*			*/*	*/*	*19*	*17.92 ± 3.55* *(2.98-70.56)*	*31*	*71.46 ± 32.59* *3.44-970.70)*		
***CA199 (U/ml)***	*/*	*/*	*22*	*2.77 ± 1.63* *0.60-36.90)*	*31*	*978.10 ± 693.60* *(0.60-20000.00)*			*/*	*/*	*19*	*20.12 ± 3.43* *(0.60-45.87)*	*28*	*17.68 ± 2.60* *4.23-62.59)*		
***CA724 (U/ml)***	*/*	*/*	*20*	*1.82 ± 0.12* *(0.82-3.20)*	*31*	*6.60 ± 2.45* *1.10-62.43)*			*/*	*/*	*19*	*6.64 ± 2.73* *(0.96-51.66)*	*32*	*30.01 ± 18.64* *(0.95-600.00)*		
***NSE (ng/ml)***	*/*	*/*	*22*	*21.98 ± 4.10* *(3.18-72.86)*	*46*	*15.88 ± 1.56* *(2.48-45.11)*			*/*	*/*	*19*	*18.85 ± 2.31* *(9.77-54.46)*	*31*	*15.83 ± 1.25* *(9.60-46.97)*		
***ProGRP*** ***(pg/ml)***	*17*	*116.40 ± 11.77* *(34.50-211.00)*	*22*	*230.10 ± 18.04* *(60.70-344.50)*	*43*	*155.30 ± 12.09* *37.20-418.30)*			*/*	*/*	*19*	*53.15 ± 4.80* *29.75-118.40)*	*35*	*45.74 ± 4.19* *1.80-123.20)*		
Validation cohort																
Tumor markers	CSF								Serum							
	Control		Wiot-LM/BrM		LM		BrM		Control		Wiot-LM		LM		BrM	
	Number	Mean ± SEM (Min-Max)	Number	Mean ± SEM (Min-Max)	Number	Mean ± SEM (Min-Max)	Number	Mean ± SEM (Min-Max)	Number	Mean ± SEM (Min-Max)	Number	Mean ± SEM (Min-Max)	Number	Mean ± SEM (Min-Max)	Number	Mean ± SEM (Min-Max)
***HE4 (pmol/L)***	*40*	*90.71 ± 6.23* *35.20-254.00)*	*19*	*91.01 ± 9.90* *50.90-204.00)*	*50*	*789.20 ± 77.63* *52.50-1500.00)*	*26*	*93.35 ± 8.25* *31.70-241.00)*	*40*	*59.22 ± 4.58* *29.70-209.00)*	*19*	*134.2 ± 27.38* *(9.83-505.00)*	*49*	*153.10 ± 29.25* *20.40-1010.00)*	*26*	*89.21 ± 10.63* *(5.00-242.00)*
***CEACAM6*** ***(ng/ml)***	*40*	*0.11 ± 0.02* *(0.03-0.95)*	*19*	*0.37 ± 0.16* *(0.03-2.83)*	*50*	*37.65 ± 4.97* *(0.3-127.10)*	*25*	*2.00 ± 0.60* *(0.05-10.56)*	*40*	*6.72 ± 0.46* *2.24-15.28.)*	*19*	*9.55 ± 2.66* *(0.69-48.49)*	*49*	*22.14 ± 2.77* *(1.26-76.95)*	*26*	*10.24 ± 3.30* *(1.09-83.02)*

### LUAD LM patients showed significantly higher CSF CEACAM6 level than patients without LM in test cohort

**Figure 3:**
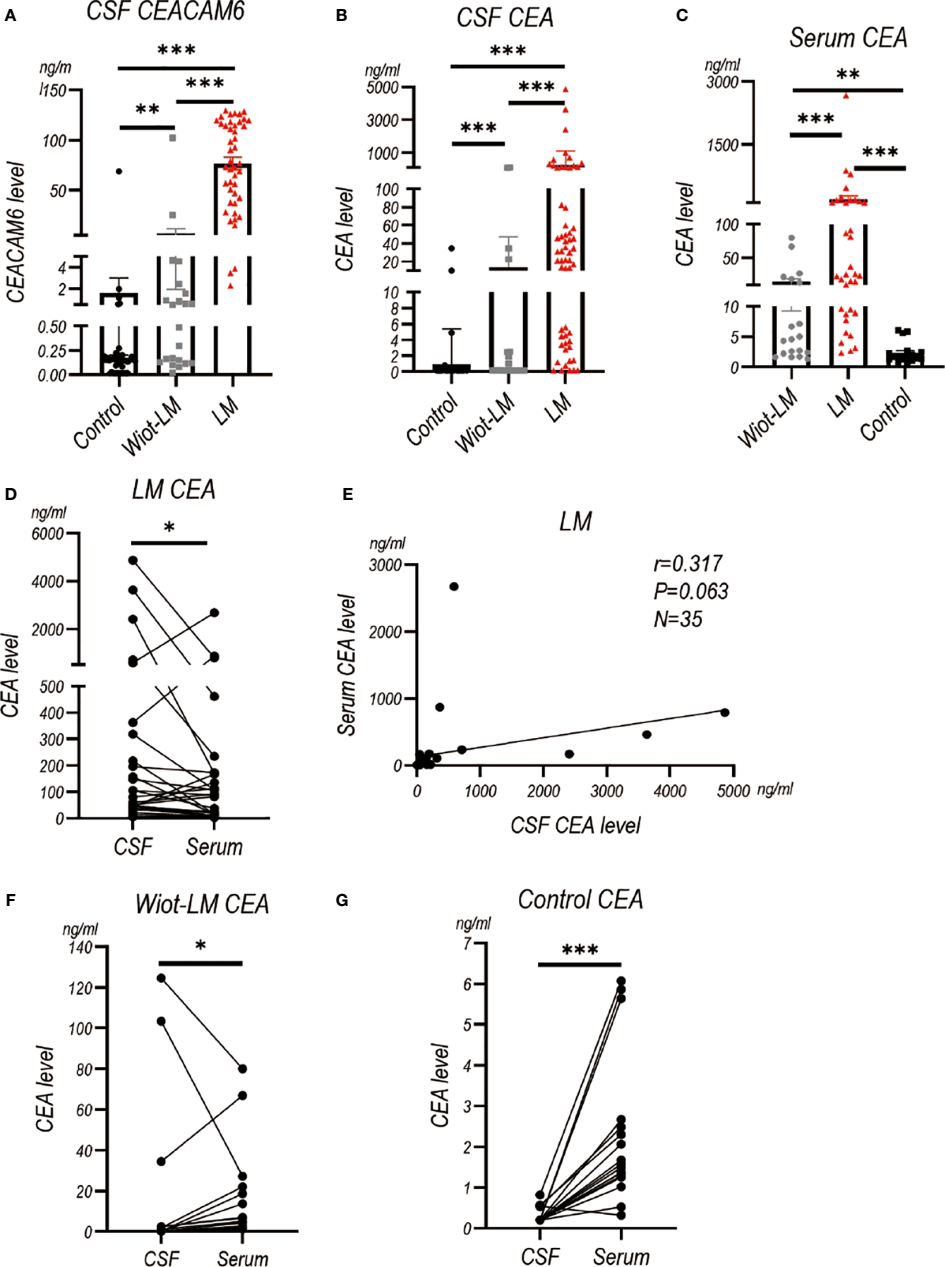
The CEACAM6 and CEA level in CSF and Serum in test cohort. **(A)** The CSF CEACAM6 level in normal samples (Control, N=49), and LUAD patients without leptomeningeal metastasis (Wiot-LM, N=22) or with LM (LM, N=45). **(B)**The CSF CEA level in Control (N=66), Wiot-LM (N=22), and LM (N=58) groups. **(C)** The serum CEA level in Control (N=17), Wiot-LM (N=19) and LM (N=35) patients. **(D)** The paired CSF and serum CEA level in LM patients (N=35). **(E)** The correlation between CSF CEA and serum CEA level in LM patients (N=35). **(F, G)** The paired CSF and serum CEA level in Wiot-LM (G, N=19) or Control group (H, N=17). ***p-value <0.001, **p-value <0.01, *p-value <0.05.

Our pervious study has showed CEACAM6 and CEA level were higher in LUAD LM than normal controls (3). Here, we enrolled Wiot-LM group and more LM patients to further support our discovery. The results showed CSF CEACAM6 level in LUAD LM patients was significantly higher than that in Wiot-LM group, whereas CSF CEACAM6 level in Wiot-LM was a little higher than that in control group (**Figure 3A**; **Table 2**).

The CEA in CSF or serum of LM group showed higher level than that in CSF or serum of Wiot-LM group (**Figures 3B, C**). The CSF CEA level was a little higher than, and had no correlation with serum HE4 level in LM patients (**Figures 3D, E**), whereas in Wiot-LM and control groups, the CEA level in CSF was lower than that in serum (**Figures 3F, G**). In this section, CSF CEACAM6 or CEA showed higher level in LUAD LM than that in Wiot-LM patients, CEA serum level would not significantly influence CSF level.

### The CSF or serum level of CA125, CA153, CA199, CA724, NSE and ProGRP in LUAD LM patients in test cohort

**Figure 4:**
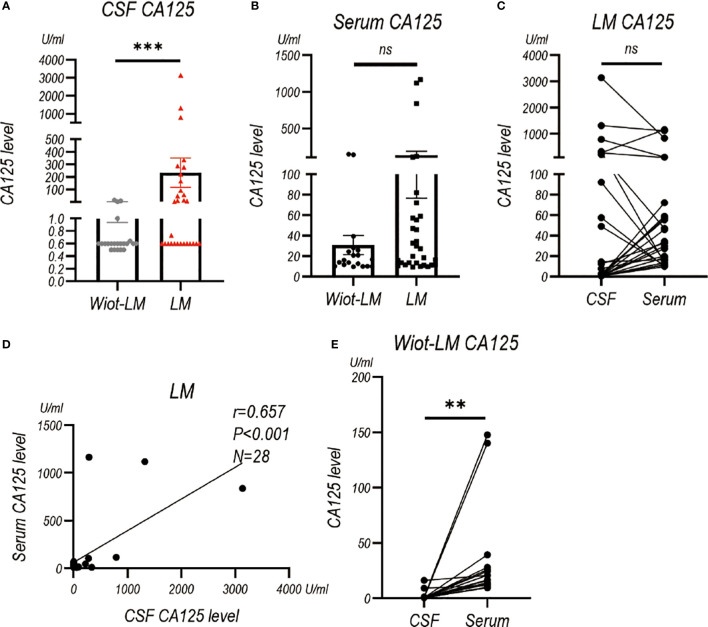
The CA125 level in CSF and Serum in test cohort. **(A)** The CA125 level in LUAD patients without leptomeningeal metastasis (Wiot-LM; N=22) or with LM (LM; N=29). **(B)** The serum CA125 in Wiot-LM (N=19) and LM (N=31) patients. **(C)** The paired CSF and serum CA125 (N=28) level in LM patients. **(D)** The correlation between CSF and serum CA125 (N=28) level in LM patients. **(E)** The paired CSF and serum CA125 (N=19) level in Wiot-LM patients. ***p-value <0.001, **p-value <0.01, ns, not statistically significant.

Considering CA125, CA153, CA199, CA724, NSE and ProGRP are common tumor biomarkers used for LUAD, we also examined their levels in CSF. CSF CA125 level (P<0.001) was higher in LM group than that in Wiot-LM group (**Figure 4A**), whereas serum CA125 showed no statistical difference between the two groups (**Figure 4B**). It is worth noting that CSF CA125 had no difference (**Figure 4C**), but positive correlation (**Figure 4D**), with serum CA125 level in LM patients, whereas for Wiot-LM patients, CSF CA125 level was lower than serum CA125 level (**Figure 4E**).

For CA153, CA199, CA724 and NSE, there were no statistically significant difference between Wiot-LM group and LM group in CSF and serum level (**Supplementary Figures 1A–D**, **2A–D**). The CA153 or CA199 level in CSF was lower than, and had a positive correlation with that in serum both in Wiot-LM and LM patients (**Supplementary Figures 1E–J**). The CSF CA724 level was a little lower than serum CA724 level, whereas CSF NSE level was a little higher than serum NSE level (**Supplementary Figures 2E, F**) in LM patients. There was no difference in Wiot-LM patients, and no correlation in LM patients between CSF and serum level for CA724 and NSE (**Supplementary Figures 2G–J**).

The CSF level of proGRP in LM patients were lower compared to that in Wiot-LM patients, but had no statistical difference with that in control group (**Supplementary Figure S3A**). The serum proGRP in LM showed no difference with that in Wiot-LM patients (**Supplementary Figure S3B**). In addition, we discovered that proGRP level in CSF had a positive correlation with (**Supplementary Figure S3C**), and was higher than that in serum both in Wiot-LM, LM and control groups (**Supplementary Figures 3D–F**).

In conclusion, in addition to the higher CSF CA125 level in LM group than that in Wiot-LM group, the diagnostic power of CSF CA153, CA199, CA724, NSE and ProGRP for LUAD LM were not ideal.

### CSF HE4 level differentiates LUAD LM from Wiot-LM or control group in test cohort

**Figure 5:**
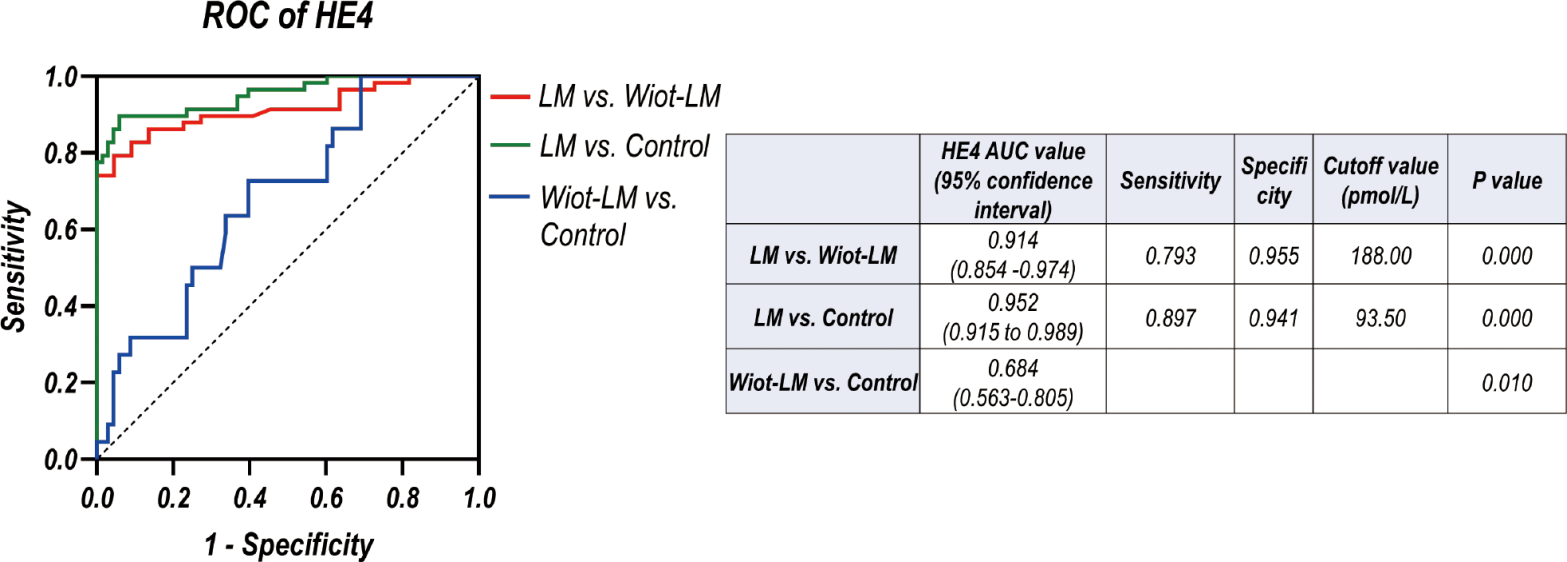
ROC curves for the CSF HE4 level for differentiating LUAD LM (N=58) from Wiot-LM (N=22) patients or controls (N=68) in test cohort.

To evaluate the diagnostic efficiency of the CSF HE4 concentration for LUAD LM, a receiver operating characteristic (ROC) curve was performed. The area under the curve (AUC) for the HE4 level was 0.914 and the confidence interval (CI) was 0.854 - 0.974 for differentiating LUAD LM from Wiot-LM patients (**Figure 5**). When the HE4 level was 188.00 pmol/L, the sensitivity was 0.793 and the specificity was 0.955. In addition, the ROC-AUC for HE4 was 0.952 for differentiating LUAD LM from controls (0.897 sensitivity and 0.941 specificity) at cut-off value of 93.50 pmol/L (**Figure 5**). CSF HE4 level displayed good performance for differentiating LUAD LM from Wiot-LM or control patients.

### Combined diagnostic value of CSF HE4 and CEACEAM6 for LUAD LM in test cohort

**Figure 6:**
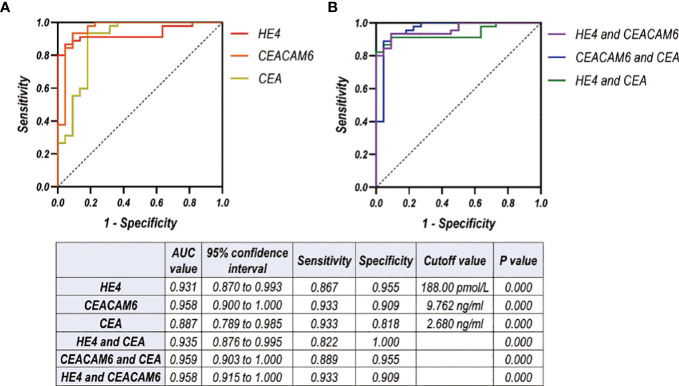
**(A, B)** ROC curves for CSF level of HE4, CEACAM6, CEA used alone **(A)** or combined with each other **(B)** for differentiating LUAD LM (N=45) from Wiot-LM (N=22) patients in test cohort.

A total of 49 samples in control group, 22 patients in Wiot-LM group, and 45 patients in LM group were enrolled in the analysis of combined diagnostic value. For differentiating LUAD LM from Wiot-LM patients, the sensitivity and specificity values were 0.867 and 0.955 at a cutoff value of 188.00 pmol/L for HE4 (P = 0.000), 0.933 and 0.909 at a cutoff value of 9.76 ng/mL for CEACAM6 (P = 0.000), 0.933 and 0.818 at a cutoff value of 2.68 ng/mL for CEA (P = 0.000). The ROC-AUC for HE4 was 0.931, for CEACAM6 was 0.958, for CEA was 0.887 respectively (**Figure 6A**).

The analysis on ROC-AUC values of the combination of two biomarkers showed that the addition of CEA did not significantly enhance AUC values of HE4 or CEACAM6 (**Figure 6B**). The better discriminative power was achieved when HE4 was combined with CEACAM6 [AUC: 0.958, (95% CI, 0.915 to 1.000), sensitivity: 0.933, specificity: 0.909] (**Figure 6B**).

The ROC analysis of other tumor markers was also performed. The AUC for CA125, CA153, CA199, CA724, NSE and ProGRP was worse than that of HE4, CEACAM6 or CEA for differentiating LUAD LM from Wiot-LM patients (**Supplementary Figure 4**).

In summary, when HE4 was used for LM diagnosis in combination with CEACAM6, the better discrimination ability was achieved.

### Confirmation of diagnostic value of CSF HE4 and CEACEAM6 for LUAD LM in an independent validation cohort

We enrolled a validation cohort to further analyze the diagnostic value of CSF HE4 and CEACEAM6. In validation cohort, the Wiot-LM group were further divided into Wiot-LM patients without brain metastases (Wiot-LM/BrM), and Wiot-LM patients with BrM (BrM) to analyze the influence of brain metastases on CSF HE4 and CEACAM6 level. In total, the validaition cohort consisted of 50 LUAD LM patients, 19 Wiot-LM/BrM patients, 26 BrM patients, and 40 normal controls.

**Figure 7:**
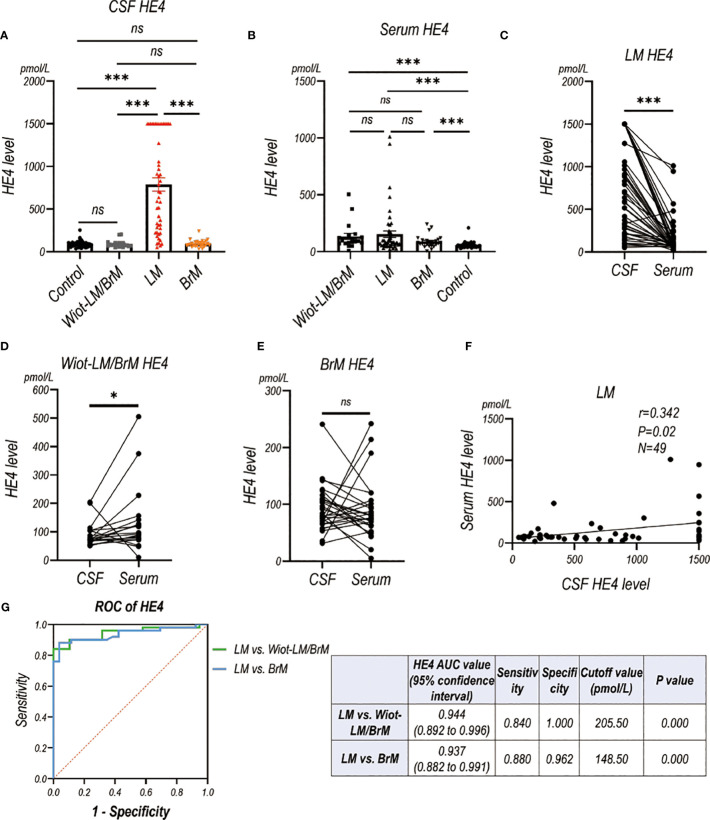
The HE4 level in CSF and Serum in validation cohort. **(A)** The CSF HE4 level in normal control samples (Control, N=40), and LUAD patients with leptomeningeal metastasis (LM, N=50), LUAD patients without LM and brain metastasis (Wiot-LM/BrM, N=19), and Wiot-LM patients with BrM (N=25). **(B)** The serum HE4 level in Control (N=40), Wiot-LM/BrM (N=19), LM (N=49) and BrM (N=25) patients. **(C)** The paired CSF and serum HE4 level in LM patients (N=49). **(D, E)** The paired CSF and serum HE4 level in Wiot- Wiot-LM/BrM (N=19) and BrM (N=25) patients. **(F)** The correlation between CSF and serum HE4 level (N=49) in LM patients. **(G)** ROC curves for the CSF HE4 level for differentiating LUAD LM (N=49) from Wiot-LM/BrM (N=19) or BrM (N=25) patients. ***p-value <0.001, **p-value <0.01, *p-value <0.05, ns, not statistically significant.

CSF HE4 level in LUAD-LM patients were significantly higher than those in the Wiot-LM/BrM, BrM and control groups, however showed no difference among Wiot-LM/BrM, BrM and control groups (**Figure 7A**). As expected, the serum HE4 level in LM, Wiot-LM/BrM, and BrM group was higher than that in normal control patients, whereas had no difference among LM, Wiot-LM/BrM, and BrM groups (**Figure 7B**). For LM patients, the HE4 level in CSF was significantly higher than that in serum (**Figure 7C**) which was not discovered in Wiot-LM and BrM group (**Figures 7D, E**). In addition, the CSF HE4 level had weak correlation with serum HE4 (**Figure 7F**) in LM group. The area under the curve (AUC) for the HE4 was 0.944 (95%CI: 0.892 to 0.996) for differentiating LUAD LM from Wiot-LM/BrM, and 0.937 (95%CI: 0.882 to 0.991) for differentiating LUAD LM from BrM (**Figure 7G**).

**Figure 8:**
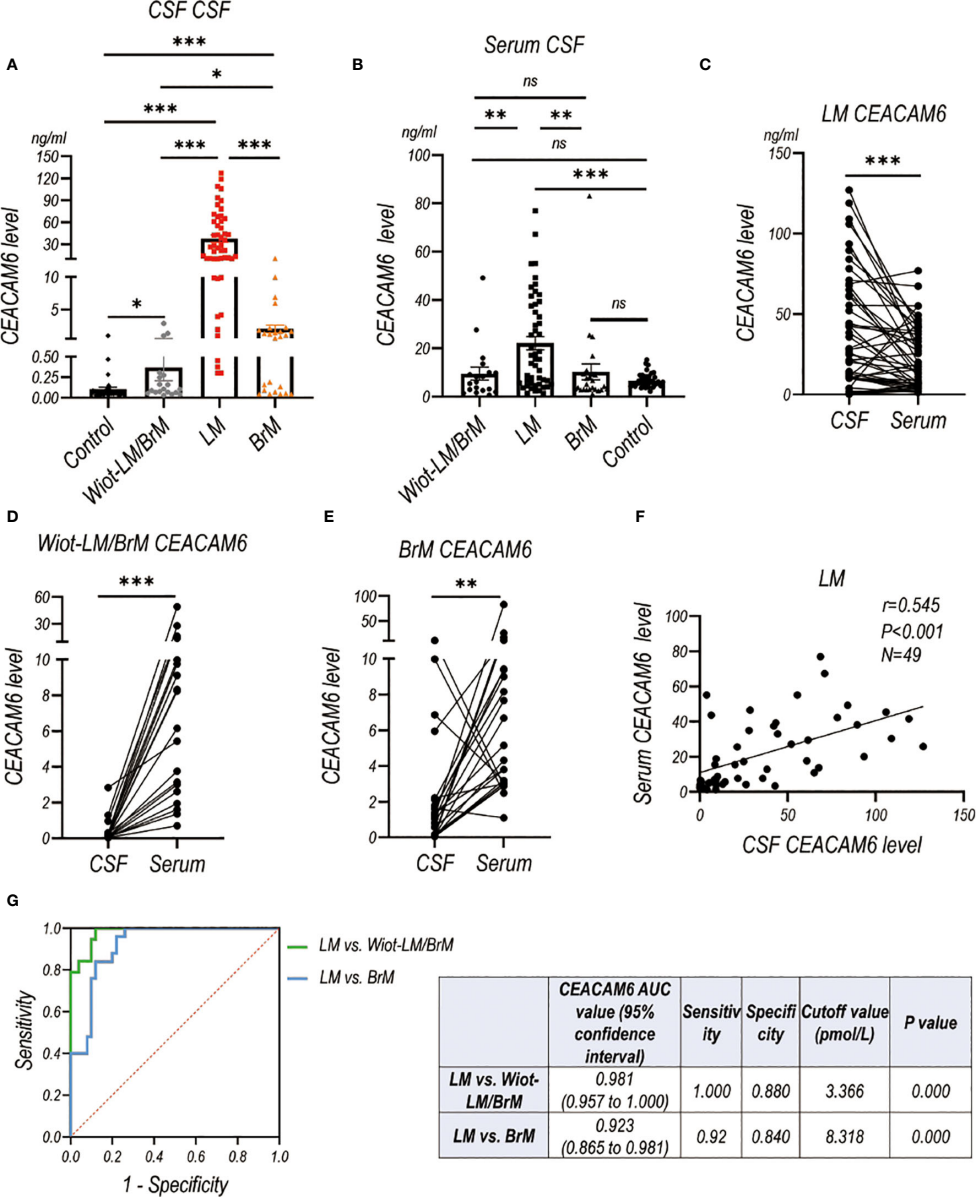
The CEACAM6 level in CSF and Serum in validation cohort. **(A)** The CSF CEACAM6 level in normal control samples (Control, N=40), and LUAD patients with leptomeningeal metastasis (LM, N=50), LUAD patients without LM and brain metastasis (Wiot-LM/BrM, N=19), and Wiot-LM patients with BrM (N=25). **(B)** The serum CEACAM6 level in Control (N=40), Wiot-LM/BrM (N=19), LM (N=49) and BrM (N=25) patients. **(C)** The paired CSF and serum CEACAM6 level in LM patients (N=49). **(D, E)** The paired CSF and serum CEACAM6 level in Wiot- Wiot-LM/BrM (N=19) and BrM (N=25) patients. **(F)** The correlation between CSF and serum CEACAM6 level (N=49) in LM patients. **(G)** ROC curves for the CSF CEACAM6 level for differentiating LUAD LM (N=49) from Wiot-LM/BrM (N=19) or BrM (N=25) patients. ***p-value <0.001, **p-value <0.01, *p-value <0.05, ns, not statistically significant.

LUAD LM patients had significantly higher CSF and serum CEACAM6 level than Wiot-LM/BrM, BrM and control patients (**Figures 8A, B**). For BrM patients, their CSF CEACAM6 level was a little higher than that in Wiot-LM/BrM patients and controls (**Figures 8A, B**). Same as the HE4, the CEACAM6 level in CSF was significantly higher than that in serum in LM patients (**Figure 8C**), whereas the result was exactly reverse in Wiot-LM/BrM and BrM group (**Figures 8D, E**). Interesting, the CSF CEACAM6 level had a positive correlation with serum CEACAM6 in LM group (**Figure 8F**). Furthermore, CSF CEACAM6 displayed good performance for differentiating LUAD LM from Wiot-LM/BrM or BrM patients, the ROC-AUC for CEACAM6 was 0.981 (95%CI: 0.957-1.000) or 0.923 (95%CI: 0.865 to 0.981) respectively (**Figure 8G**).

In general, we further confirmed that CSF HE4 and CEACAM6 level in LM groups were significantly higher than those in Wiot-LM (Wiot-LM/BrM and BrM) and control groups in the validation cohort. BrM did not influence the CSF HE4 and CEACAM6 levels observably. In addition, CSF HE4 and CEACAM6 have high sensitivity and specificity when distinguishing between LM and Wiot-LM/BrM or BrM.

**Figure 9:**
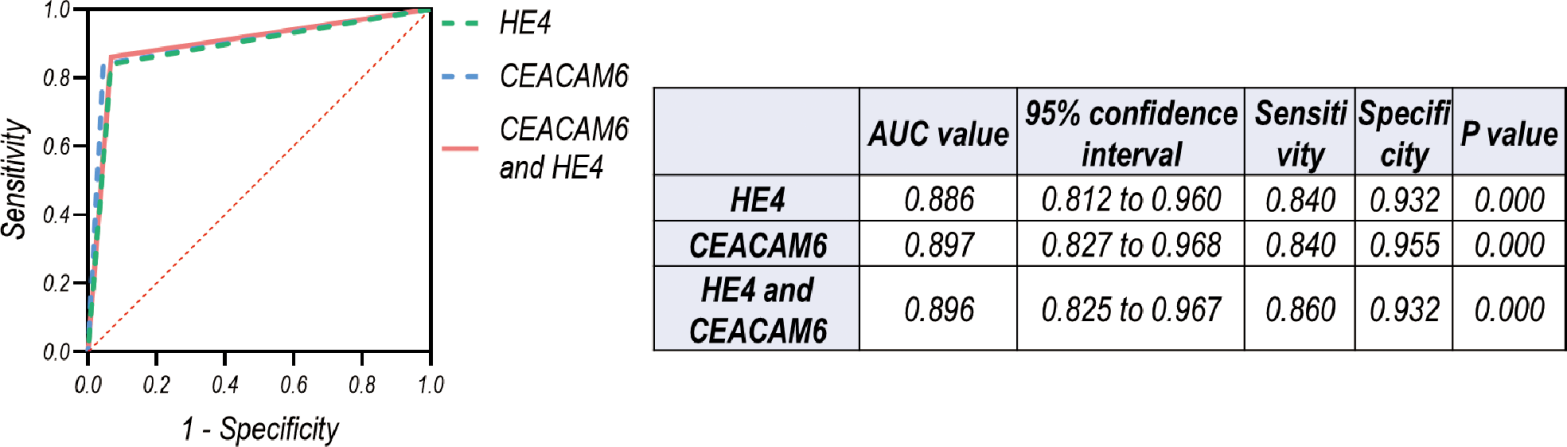
ROC curves for CSF level of HE4 and CEACAM6 for differentiating LUAD LM (N=50) from Wiot-LM (N=44) patients in validation cohort when applying the cutoff value defined in test cohort (**Figure 6**).

The cutoff value for HE4 was 188.00 pmol/L, for CEACAM6 was 9.76 ng/mL when differentiating LUAD LM from Wiot-LM (Wiot-LM/BrM and BrM) patients in the test cohort, which were applied in validation cohort to perform ROC analysis (**Figure 9**). The results showed sensitivity and specificity values for HE4 were 0.840 and 0.932 (ROC = 0.886), for CEACAM6 0.840 and 0.955 (ROC = 0.897), for the combination of HE4 and CEACAM6 were 0.860 and 0.932 (ROC=0.896). The validation cohort further confirmed the diagnostic value of CSF HE4 and CEACEAM6 in LUAD LM.

## Discussion

CSF tumor biomarkers for LM had the advantage of technical simplicity and low cost. It is easier to adopt widely in clinical practice. CSF tumor markers helping the diagnosis of LM are urgently required. Our previous study has defined the CEACAM6 as a tumor biomarker for LM, but the control group were normal CSF samples from benign disease patients, not LUAD patients without LM (Wiot-LM). Here, we enrolled Wiot-LM patients in two different cohorts to further support CEACAM6 diagnostic efficiency for LUAD LM patients. As expected, the high diagnostic efficacy of CSF CEACAM6 was defined and superior to that reported in previous study (3). The CSF samples collected from LUAD LM patients in the study all displayed positive cytology, whereas in previous study, some CSF samples of LUAD LM patients showed negative cytology due to therapy. CSF-CTCs had positive influence on CSF CEACAM6 level. However, in this study, the CSF CEACAM6 level in LM patients detected by ELISA was significantly higher than that in previous study (3), which may attributable to different batches of ELISA KIT. It is necessary to establish standardized methods for CEACAM6 testing in clinic practice. In conclusion, the study further confirmed the superior diagnostic performance of CSF CEACAM6 for LUAD LM patients.

In addition to diagnostic value of serum and pleural fluid, HE4 level in the urine and other body fluids were also confirmed. HE4 detection in urine, saliva, and tear offered a noninvasive test for tumor of screening, therapeutic efficacy monitoring and recurrence (12). In bronchial aspiration fluid, HE-4 value showed no significant difference between lung cancer and benign lung disease (13). Umbilical cord HE-4 level was statistically higher in term deliver group than preterm delivery group, and umbilical cord HE-4 could predict fetal lung maturity (14). Here, the study indicated that CSF HE4 displayed good diagnostic efficiency for LUAD LM. Furthermore, CEACAM6 effectively improved the diagnostic efficiency of HE4, whereas CEA supported weakly. Interestingly, we discovered that CSF HE4 level was significantly higher than serum HE4 level in LM patients, which was not discovered in Wiot-LM and controls groups. In addition, there was no correlation between CSF and serum HE4 in LM patients. It highlights the possibility that the high HE4 level in CSF of LM patients may derived from CSF tumor cells.

Although HE4 expression has been reported upregulated in various tumors, its functional roles have remained largely unknown. HE4 promotes proliferation, angiogenesis, metastatization, immune response in ovarian cancer (5). In endometrial cancer, HE4 contributes to proliferation, colony formation, and invasion (15). HE4 suppresses prostate cancer metastasis by inactivating EGFR/AKT/GSK3B/Snail signaling pathway (16). In addition, WFDC2−/− deletion C57 mouse leads to severe dyspnea and type-I alveolar cell apoptosis (17). HE4 promotes cystic pulmonary fibrosis associated inflammation by NF-κB and MAPK signalings (18). The dysfunction of cystic fibrosis transmembrane conductance regulator (CFTR) gene contributes to abnormal HE4 expression via NF-κB in cystic fibrosis (19). However, few studies have reported the roles and molecular mechanisms of HE4 in lung cancer development which deserves further exploration.

Neurological, radiographic, and CSF examination are important basis for LM diagnosis. European Association of Neuro-Oncology-European Society of Medical Oncology (EANO-ESMO) group classifies LM as type I (positive CSF cytology) or type II (negative or equivocal CSF cytology) which is supported by neurological symptoms and typical radiographic features (20). Notably, in addition to 20% of LM individuals showing type II (21), most LM patients are classified as type I. However, the sensitivity of CSF cytology is as low as 45%-67% in initial CSF analysis. Therefore, new technology liquid biopsy is emerging, such as CTCs and circulating cell-free tumor DNA (ctDNA), which can be useful for diagnosis, genetic mutations detection, and monitoring therapy responses in LM, and deserves to be update in the guidelines (22). In addition, numerous CSF tumor markers have been explored to aid in LM diagnosis, including CEA, PSA, CA15-3, CA125, CA199, AFP, NSE, Cyfra 21-1, MART-1, and MAGE-3 in melanoma (22), however their roles in clinical practice are limited. Here, we also conducted the diagnostic efficiency of common tumor biomarkers CA125, CA153, CA199, CA724, NSE and ProGRP. The diagnostic power of CA153, CA199, CA724, and NSE for LUAD LM were not ideal. The higher level of CA125 was detected in CSF of LM than Wiot-LM patients. The ROC-AUC of CA125 for distinguishing LM from Wiot-LM was worse than that of HE4 or CEACAM6. The ProGRP level in CSF has been unclear. In the study, ProGRP level was higher in CSF than that in serum both in Wiot-LM, LM and control groups. It is worth noting that CSF ProGRP level in LM was lower than that in Wiot-LM patients, but had no difference with that in control group. The diagnostic values of ProGRP for LUAD LM was confused and needed enroll more patients to validate. In this study, our results suggested the feasibility and superiority of HE4 and CEACAM6 as CSF biomarkers for LM-LUAD diagnosis. CSF tumor markers evaluation may offer reliable means to diagnose and monitor LM.

The limitations of our study. Firstly, we did not explore the impacts of different treatment modalities. After a series of treatment, if the LM patients was in remission or suffered relapses, how did the CSF CEACAM6 and HE4 level fluctuate. CSF CEACAM6 and HE4 level had the high possibility of clinical usefulness in monitoring tumor burden and clinical efficacy, and evaluating prognosis of LM patients. Second, we paid attention to the LM patients diagnosed by positive CSF cytology, whereas the HE4 and CEACAM6 levels in CSF from LM patients who were diagnosed based on clinical findings and typical radiographic features and showed negative or equivocal CSF cytology, have been still unknown. Third, whether LM patients derived from other solid tumors, such as breast cancer and melanoma representing most common causes of LM, showing positive CSF HE4 and CEACAM6 detection deserve our further attention. Finally, CSF CEACAM6 quantification was not a clinically approved method, we are establishing chemiluminescent immunoassay for clinical CEACAM6 detention.

## Conclusions

In contrast to radiographic and CSF examinations which need experienced technicians and have the subjectivity, tumor markers are easily measurable by hospital laboratories via commercially available assay kits. In the study, we confirmed that CSF HE4 functioned as a potential marker for LUAD LM for the first time. The better discriminative power was achieved when HE4 was combined with CEACAM6. CSF biomarkers HE4 and CEACAM6 may facilitate and complement the diagnosis, prognosis and clinical managing of LUAD LM in the future.

## Data availability statement

The original contributions presented in the study are included in the article/**Supplementary Material**. Further inquiries can be directed to the corresponding authors.

## Ethics statement

The studies involving humans were approved by the Institute Research Ethics Committee of the Cancer Center of Huashan hospital. The studies were conducted in accordance with the local legislation and institutional requirements. The participants provided their written informed consent to participate in this study.

## Author contributions

XL: Formal analysis, Methodology, Software, Writing – original draft. KC: Data curation, Writing – review & editing, Validation. JL: Validation, Writing – review & editing. XT: Validation, Writing – review & editing. HR: Writing – review & editing, Data curation, Funding acquisition, Project administration, Writing – original draft. MG: Funding acquisition, Project administration, Writing – review & editing.
