# Supplementary material for: Diagnostic value of cerebrospinal fluid human epididymis protein 4 for leptomeningeal metastasis in lung adenocarcinoma
Source: Front Immunol. 2024 Jan 18;15:1339914. doi: 10.3389/fimmu.2024.1339914 (PMC10830695; doi:10.3389/fimmu.2024.1339914)
Supplement: Supplementary file 1 [file DataSheet_1.doc]

*
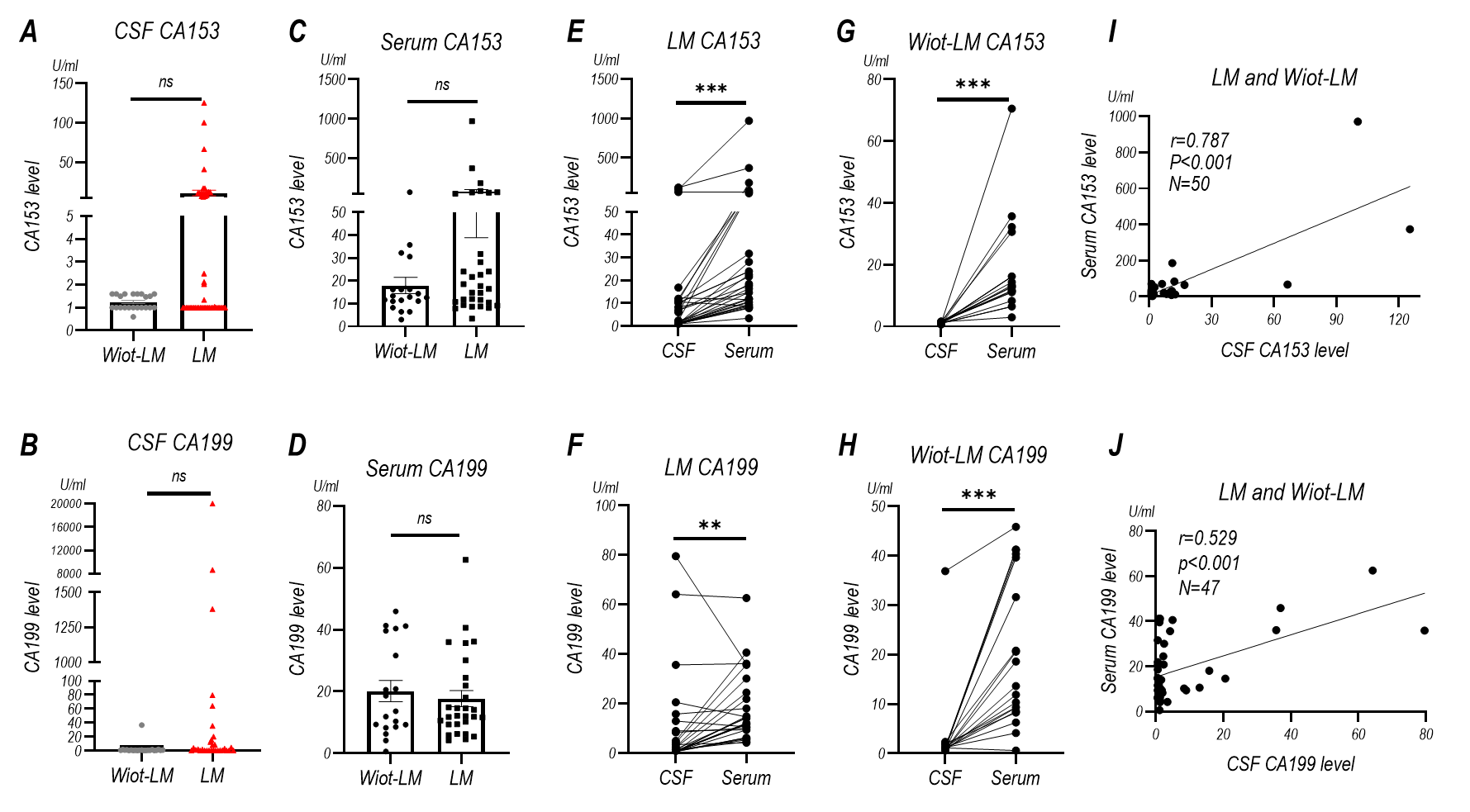
*

Figure S1. The CA153 and CA199 level in CSF and Serum in test cohort.

(A-B) The CA153 (A) or CA199 (B) level in LUAD patients without leptomeningeal metastasis (Wiot-LM; N=22) or with LM (LM; CA153, N=47; CA199, N=31). (C-D) The serum CA153 (C) or CA199 (D) level in Wiot-LM (N=19) and LM (CA153, N=31; CA199, N=28) patients. (E-F) The paired CSF and serum CA153 (E, N=31) or CA199 (F, N=28) level in LM patients. (G-H) The paired CSF and serum CA153 (I, N=19) or CA199 (J, N=19) level in Wiot-LM patients. (I-J) The correlation between CSF and serum CA153 (G, N=50) or CA199 (H, N=47) level in both LM and Wiot-LM patients. ***p-value <0.001, **p-value <0.01, ns, not statistically significant.


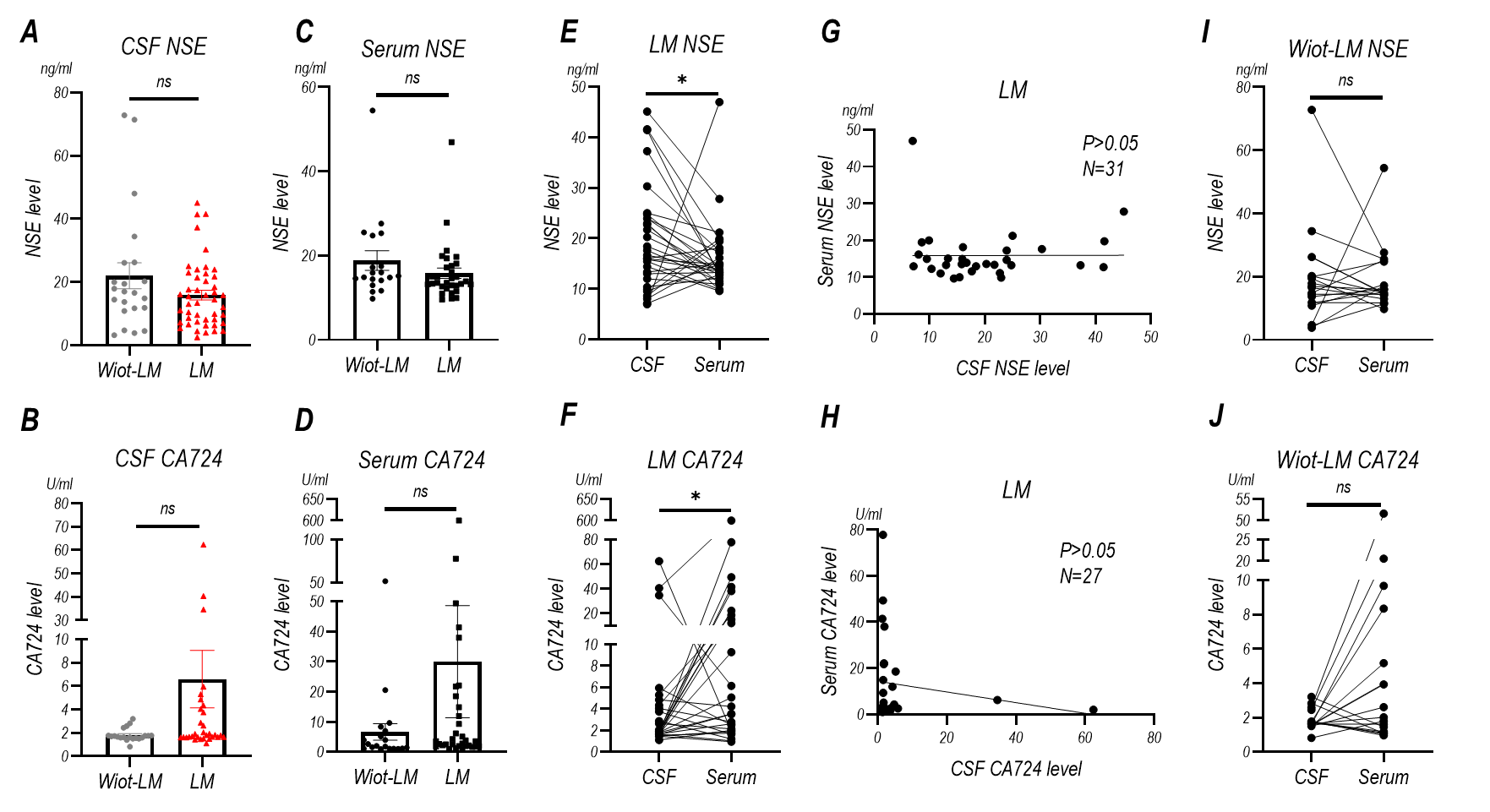


Figure S2. The NSE and CA724 level in CSF and Serum in test cohort.

(A-B) The NSE (A) or CA724 (B) level in LUAD patients without leptomeningeal metastasis (Wiot-LM; NSE, N=22; CA724, N=20) or with LM (LM; NSE, N=46; CA724, N=31). (C-D) The serum NSE (C) or CA724 (D) level in Wiot-LM (N=19) and LM (NSE, N=31; CA724, N=32) patients. (E-F) The paired CSF and serum NSE (E, N=31) or CA724 (F, N=28) level in LM patients. (G-H) The correlation between CSF and serum NSE (G, N=31) or CA724 (H, N=27) level in LM patients. (I-J) The paired CSF and serum NSE (I, N=19) or CA724 (J, N=17) level in Wiot-LM patients. *p-value <0.05, ns, not statistically significant.

*
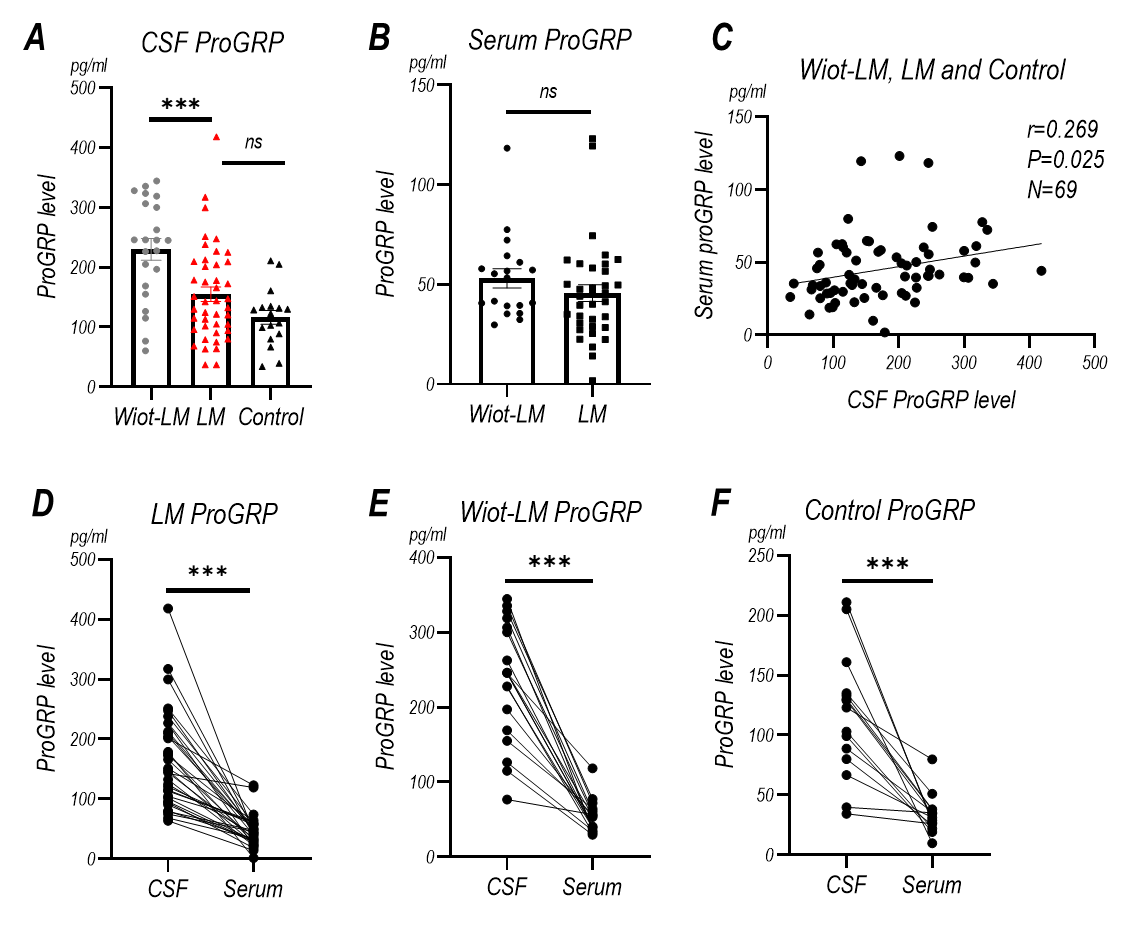
*

Figure S3. The ProGRP level in CSF and Serum in test cohort.

(A) The CSF ProGRP level in control (N=17), Wiot-LM (N=22) and LM (N=43) groups. (B) The serum ProGRP in Wiot-LM (N=19) and LM (N=35) patients. (C) The correlation between CSF and serum ProGRP (N=69) level in both LM and Wiot-LM and control groups. (D-F) The paired CSF and serum ProGRP in LM (D, N=35), Wiot-LM (E, N=19) and control (F, N=15) groups. ***p-value <0.001, ns, not statistically significant.

***
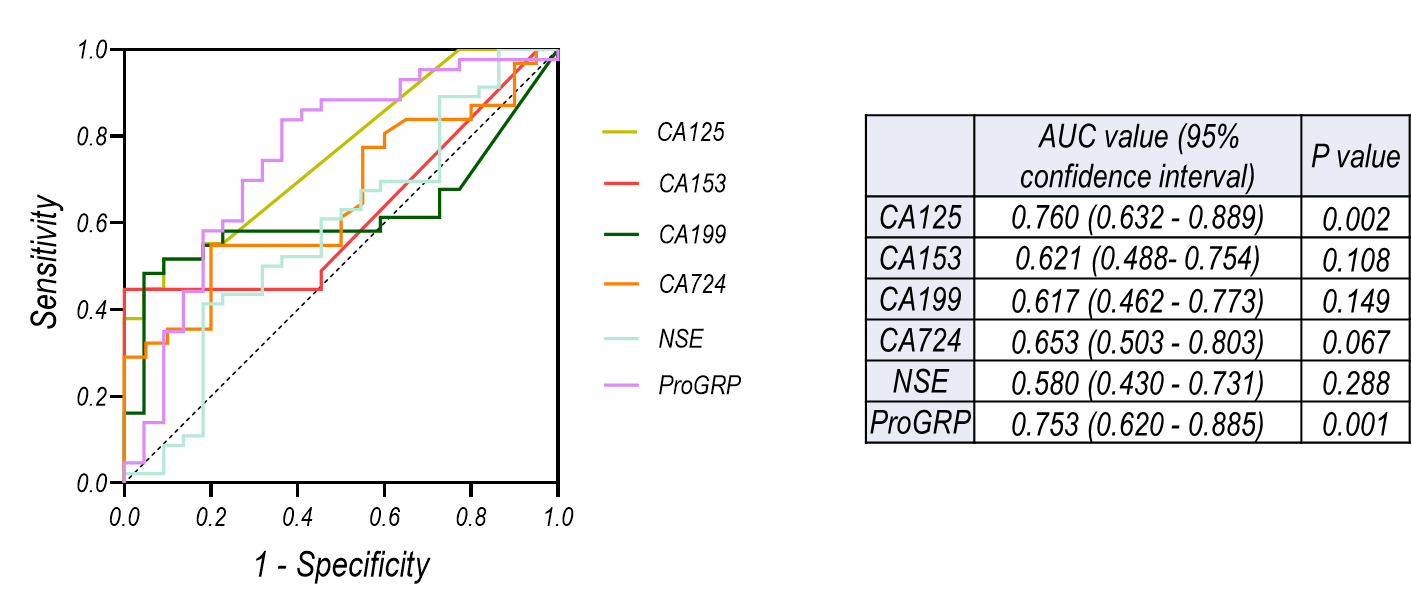
***

Figure S4. ROC curves for CSF level of CA125, CA153, CA199, CA724, NSE and ProGRP level for differentiating LUAD LM from Wiot-LM patients.
